# Isolated Recurrence of Diffuse Large B-Cell Lymphoma in Sciatic Nerve

**DOI:** 10.14740/wjon736w

**Published:** 2014-06-25

**Authors:** Chenthuran Deivaraju, Jose Francisco Inzunza, Nathan Hammel, Sheila Ann Conway

**Affiliations:** a Department of Orthopedics, University of Minnesota, Minneapolis, USA; b Westchester Medical Center, New York, USA; c University of Miami Hospital, Miami, Florida, USA

**Keywords:** Sciatica, Nerve, Non-Hodgkin’s lymphoma

## Abstract

Sciatica is a common clinical presentation with a number of etiological factors. Many of them are innocuous like prolapsed intervertebral disc or peripheral compression in the sciatic nerve. Occasionally the cause could be of a more serious nature like a nerve sheath tumor or more infrequently, lymphomatosis. We describe recurrent lymphoma in a patient who had been in remission presented with sciatica as result of the involvement of the nerve with metastatic tumor.

## Introduction

Neurolymphomatosis (NL) is an uncommon presentation of lymphoma in which there is an infiltration of cranial nerves, spinal roots, or peripheral nerves by lymphoma cells in the setting of a known or unknown hematologic malignancy [1]. It is in fact a rare neurologic presentation of non-Hodgkin’s lymphoma or leukemia. The disease could be classified as primary NL, when NL is first manifestation of a systemic disease or secondary NL when the disease relapses or recurs in nervous tissue. The involvement of a single peripheral nerve is a rare subset of presentations of NL. Despite the rarity of these cases, when they do present, the sciatic nerve is the most common site involved [1]. Here we present the case of a 24-year-old female who had a relapse of diffuse large B-cell lymphoma (DLBCL) in the sciatic nerve after being in clinical and radiographic remission following chemotherapy.

## Case Report

A 23-year-old female was diagnosed with DLBCL in October 2010. PET scan had shown that her disease was based in the mediastinum and was characterized by bulky lymphadenopathy. The patient had experienced back pain secondary to the mediastinal disease. She immediately began chemotherapy with the CHOP-R protocol. Completion of the protocol in February 2011 resulted in full resolution of her symptoms. Follow-up PET imaging demonstrated no evidence of lymphoma.

**Figure 1:**
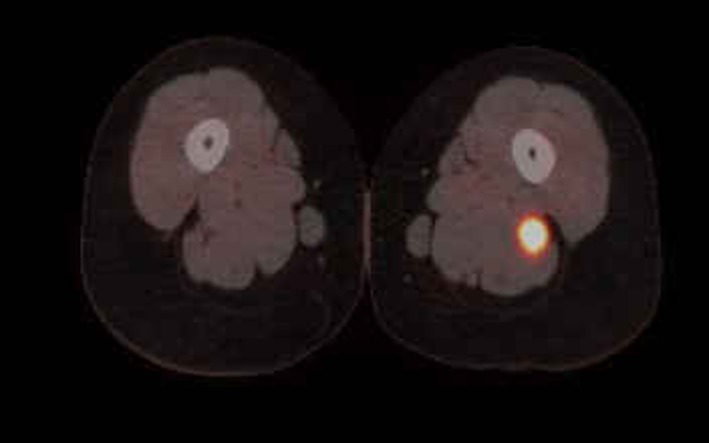
PET/CT clearly shows enlargement and increased uptake in the axial section.

**Figure 2:**
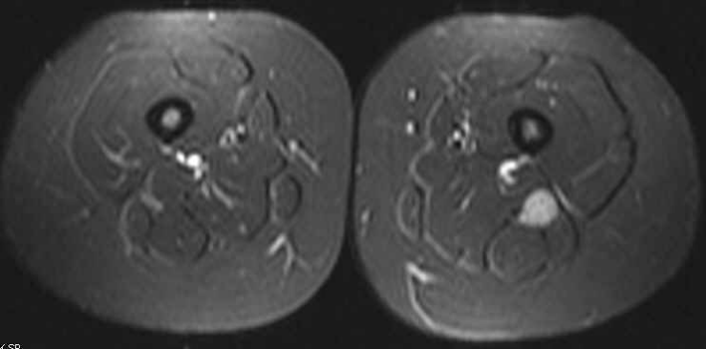
T2-weighted image showing enlargement of the sciatic nerve when compared to the other side.

In June 2011, a surveillance PET/CT scan revealed a tubular-shaped density within the left thigh (Fig. 1). MRI evaluation of the mass demonstrated a spindle-shaped structure involving the sciatic nerve (Fig. 2). Around the same time, the patient began to experience symptoms of burning sensations and numbness and tingling in the toes of her left foot and left lower leg paresthesias in the region of the common peroneal nerve after prolonged sitting. Her physical exam demonstrated a positive straight leg raise test on the left with exacerbation of her symptoms. An ultrasound-guided biopsy was performed, revealing DLBCL cells with infiltration of the sciatic nerve. The perineurium, the endoneurim and the nerve fibers were identified; between them were large lymphoid cells that looked identical to the original lymphoma cells. An immunohistochemical stain showed that all of these large cells were CD20 positive. The histology and immunohistochemical staining were diagnostic of lymphoma of the sciatic nerve.

## Discussion

It has been shown that up to 40% of DLBCLs present with extranodal disease, and that the vast majority of NL cases are B-cell lymphomas [2]. However, there are instances where the invading cells were of the T-cell lineage [3, 4]. Additionally, almost any extra nodal location can be a primary site [2]. Primary NL affecting the sciatic nerve has been reported several times in the literature [5]. Occasionally, lymphomas are seen in peripheral nerves as a recurrence to a primary in a distant nerve [5]. By all accounts, direct invasion of the PNS by lymphoma, whether an initial or as a recurrent presentation, is rare [5]. In cases of relapsed lymphoma that presents in a peripheral nerve, it is believed that the blood-nerve barrier is penetrable to lymphoma cells, but not to chemotherapy, thus providing a safe haven for a recurrence to occur [6].

Common presentations of NL include painful peripheral neuropathy, painful radiculopathy, cranial neuropathy or a painless polyneuropathy [7]. Presenting symptoms have been reported such as foot drop in one patient [8] and large toe numbness in another [9]. All of these patients, including ours, had symptoms that quickly evolved into a presentation of acute sciatica. A progressive sciatic neuropathy is highly suspicious for a neoplasm. The most common causes are schwannoma or neurofibroma, but NL is also in the differential diagnosis [10]. NL should be high on the list of the differential particularly when there is a history of non-Hodgkin’s lymphoma. The median time for a recurrence to occur is 10 months [7]. However, the recurrence could occur years after the primary disease was treated. Clinically, patients will typically have findings of severe pain, asymmetric distribution and rapid evolution. MRI has been shown to have some utility in the diagnosis of NL, demonstrating abnormal enhancement of the affected neural structure, which show up as thickened, diffuse or nodular [7]. These MRI findings are nonspecific for NL, and are also observed in inflammatory radiculopathies or neuropathies, neurofibromatosis, and benign and malignant nerve sheath tumors [7]. However, radiological findings of large lesion size and homogeneous contrast uptake are not usually seen in tumors of neural origin, such as neural sheath tumors, which make PNS lymphoma a more likely etiology [1]. It should be utilized along with electrophysiological studies in all cases of atypical sciatica [11].

### Conclusion

Due to NL having associations with a history of non-Hodgkin’s lymphoma or leukemia, patients presenting with a mono- or poly-neuropathy should be worked up for in intraneural tumor.
